# Ultrasensitive Wearable Strain Sensors of 3D Printing Tough and Conductive Hydrogels

**DOI:** 10.3390/polym11111873

**Published:** 2019-11-13

**Authors:** Jilong Wang, Yan Liu, Siheng Su, Junhua Wei, Syed Ehsanur Rahman, Fuda Ning, Gordon Christopher, Weilong Cong, Jingjing Qiu

**Affiliations:** 1 Key Laboratory of Textile Science & Technology of Ministry of Education, College of Textiles, Donghua University, Shanghai 201620, China; jilong.wang@dhu.edu.cn (J.W.); yan.liu@mail.dhu.edu.cn (Y.L.); 2 Department of Mechanical Engineering, California State University Fullerton, Fullerton, CA 92831, USA; ssu@fullerton.edu; 3 Department of Mechanical Engineering, Texas Tech University, 2500 Broadway, Lubbock, TX 79409, USA; junhua5wei@gmail.com (J.W.); syed.rahman@anton-paar.com (S.E.R.); gordon.christopher@ttu.edu (G.C.); 4 Department of Systems Science and Industrial Engineering, State University of New York at Binghamton, Binghamton, NY 13902, USA; fning@binghamton.edu; 5 Department of Industrial Engineering, Texas Tech University, 2500 Broadway, Lubbock, TX 79409, USA; weilong.cong@ttu.edu

**Keywords:** hydrogels, 3D printing, tough, sensor

## Abstract

In this study, tough and conductive hydrogels were printed by 3D printing method. The combination of thermo-responsive agar and ionic-responsive alginate can highly improve the shape fidelity. With addition of agar, ink viscosity was enhanced, further improving its rheological characteristics for a precise printing. After printing, the printed construct was cured via free radical polymerization, and alginate was crosslinked by calcium ions. Most importantly, with calcium crosslinking of alginate, mechanical properties of 3D printed hydrogels are greatly improved. Furthermore, these 3D printed hydrogels can serve as ionic conductors, because hydrogels contain large amounts of water that dissolve excess calcium ions. A wearable resistive strain sensor that can quickly and precisely detect human motions like finger bending was fabricated by a 3D printed hydrogel film. These results demonstrate that the conductive, transparent, and stretchable hydrogels are promising candidates as soft wearable electronics for healthcare, robotics and entertainment.

## 1. Introduction

As we know, human skin is soft, self-healable and stretchable, and has the ability to sense subtle external changes. This amazing property has attracted tremendous interest in artificial skin, especially wearable electronics for healthcare, artificial intelligence and soft robotics [1,2,3]. These artificial skin-like devices can monitor environmental stimuli such as pressure, strain, temperature, and deformation by detecting electric signals like current and voltage, or measuring electrical properties including resistance, and capacitance. “Electronic skin” is usually considered as a stretchable sheet with area above 10 cm^2^ integrating sensors to detect different external stimuli [4]. Usually, electronic skin is made of stretchable electrical conductors including carbon grease [5], graphene sheets [6], carbon nanotubes [7], liquid metals [8] and metal nanostructures [9,10], which transmits signals via electrons. Although these materials present high conductivity and excellent stretchability, which meet the necessary requirements of electronics skin, they fail to meet other additional requirements like biocompatibility and transparency. On the other side, human skin can report signals via ions, which provides a potential pathway to develop ionic conductors based on a sensory sheet called “ionic skin” [4]. Hydrogels are three-dimensional networks composed of high-molecular weight polymer, large amounts of water, and crosslinkers [11]. As the water in hydrogels can dissolve ions, hydrogels can be employed as ionic conductors [4,12], which may have potential applications in “ionic skin”. In addition, hydrogels are highly stretchable and biocompatible [13]. Furthermore, high transparency of hydrogels allows these sensory sheets to report electrical signals without impeding optical signals [4]. They can behave as tough as elastomers due to recent developments [14,15,16,17], which can monitor large deformation, like finger bending.

Three-dimensional (3D) printing, also known as an additive manufacturing process, is an emerging technology [18]. Due to its rapid production with high shape fidelity, 3D printing technology has attracted tremendous attention since it was first proposed by Charles W. Hull in 1986 [19]. Recently, volumetric additive manufacturing has been developed and has received lots of attention due to its excellent performance to overcome limitations of low speed and geometric constraints [20,21,22]. However, the 3D printing technology has been recently introduced to fabricate hydrogels. Extrusion printing method is a modified fused deposition modeling method that extrudes continuous liquid inks to achieve layered structures. As the extrusion printing method has lots of advantages including simple fabrication procedure, large range of materials, good balance between printer’s cost and printing quality, and high cell deposition in bioprinting, it has been considered as an excellent choice to print hydrogels [17,23].

Various polymers have relatively high viscosity to maintain their pattern in printing process, and have crosslinking abilities allowing for 3D structures maintenance after printing, like collagen [24], hyaluronic acid (HA) [25], chitosan [26] and alginate [18], have been employed in 3D printing technology to achieve 3D printed hydrogels. Usually, physical crosslinking can be induced by temperature change [15,24] and ionic crosslinking [11,27], whereas chemical crosslinking can be formed by polymerization [28]. Sodium alginate (SA), an anionic polymer isolated from brown algae, has the ability to crosslink assisting by divalent or trivalent ions [11]. Due to its high biocompatibility, hydrophilicity and biodegradability under normal physiological conditions, SA has received increasing attention as an instant gel for tissue engineering. Although there are lots of conventional methods to fabricate SA hydrogel constructs including the injection molding method and solution casting method, SA solution has a certain viscosity and its limited flowability leads to a poor dispersion in molds [11]. Compared to these conventional methods, 3D printing technology has one main advantage to fabricate customized constructs, which can fit various requirements of wearable sensors applied on different body parts on different humans [17]. In addition, 3D printing technology has the potential to fabricate hydrogels with hierarchically porous structures or gradient properties, which may improve sensitivity and sensing range of wearable sensors [29]. Although 3D printing technology has these advantages to fabricate SA hydrogel constructs, several challenges have not been well addressed, which limits its development. One of the common challenges of 3D printing hydrogels is to achieve printed constructs with high shape fidelity due to low viscosity leading to a collapse tendency of the printed constructs. Various methods have been proposed to increase the viscosity of SA solution, such as increasing the SA ink concentration or varying molecular weight [30], combining with other materials including nanocellulose [18] or gelatin [31], employing a supporting sacrificial polymer [32] and partially crosslinking alginate with calcium ions [33].

To improve the printing resolution and mechanical property, the hybrid agar/calcium alginate (CA)/polyacrylamide(PAAm) hydrogels combining brittle thermo-responsive agar and ionic-responsive alginate and soft polyacrylamide network is proposed in this manuscript. During printing, mixture of thermo-responsive agar and ionic-responsive alginate was extruded as a continuous stripe to enhance printing resolution. Meanwhile, due to the increasing viscosity, the mixture can maintain its shape during printing. After photopolymerization and solution soaking, the 3D printed tough hydrogels were achieved. With calcium crosslinking of alginate, tensile strength and fracture energy of 3D printed hydrogels are greatly improved. Furthermore, the water in 3D printed hydrogels dissolves lots of calcium ions, which make them work as ionic conductors. A wearable soft resistive strain sensor was developed by a 3D printed hydrogel film. This resistive strain sensor exhibits quick and accurate detection of changes of finger bending, which demonstrates that the conductive, transparent, stretchable hydrogels can be used as wearable resistive strain sensor to monitor human motion.

## 2. Experimental Section

### 2.1. Materials

Sodium alginate was received from FMC biopolymer (Rockland, ME, USA). Agar, acrylamide, *N*,*N*’-methylenebis (acrylamide) (MBAA), Irgacure 2959 and calcium chloride were ordered from Sigma-Aldrich (St. Louis, MO, USA) without further purification. An acrylic elastomer (VHB 4905) was received from 3M (St. Paul, MN, USA). Copper tape was used to connect conductive gels and electric wires.

### 2.2. 3D Printing System

**Figure 1:**
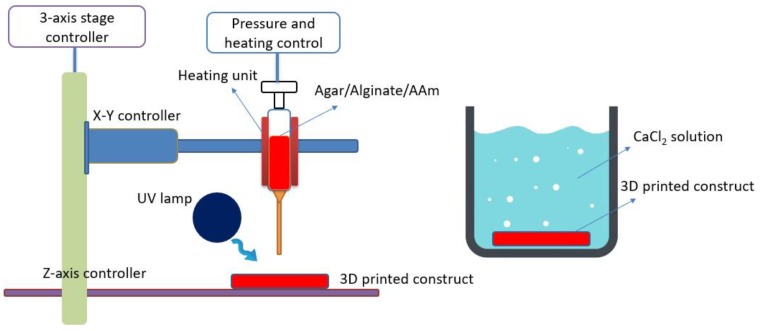
Schematic diagram of a 3D printing procedure.

As shown in Figure 1, a modified Leapfrog 3D printer was employed to fabricate tough hydrogels as described in our previous literature [18]. In brief, a syringe pump (NE-500 OEM, New Era, Gawler, Australia) was installed onto 3D printer to extrude pre-gel ink at a controlled infusion velocity (0.73 µL h^−1^–2100 mL h^−1^), and a thermal pad (HEATER-KIT-5SP, New Era, Gawler, Australia) was used to wrap up syringe to maintain printing temperature. The blunt tip needles (gauge 14–26) were used to inject continuous hydrogel solution. A commercial software “Simplify 3D” was applied to control printing process. 

### 2.3. 3D Printing Hydrogels Fabrication

The 3D printing procedure was performed on a modified Leapfrog 3D printer that is similar to our previous literature [17]. SA 200 mg was first dissolved in 10 mL DI water with continuous stirring overnight. Then the SA solution was heated to 95 °C in an oil bath, and 200 mg of agar was added into the solution. After agar was fully dissolved in the water and 3000 mg acrylamide and corresponding MBAA, Irgacure 2959 were added, the hybrid ink was cooled to 55 °C and ready for printing. As shown in Figure S1, three different infill angles were used to achieve 3D printing constructs. The width and length of design were set at 100 mm. The thickness of 3D printing constructs was around 1 mm. After printing, the printed construct was exposed to UV light (365 nm) for 1 h. Then 100 mM CaCl_2_ solution was used to crosslink sodium alginate for 15 min. To achieve conductive printed hydrogels, the printed gels was soaking in 100 mL 100 mM CaCl_2_ solution overnight to increase the concentration of Ca^2+^ ions in printed gels. These excess Ca^2+^ ions can be used as ions carriers. This sample was labelled as A2C2. The formula and labels of other samples were summarized in Table 1.

### 2.4. Mechanical Test

The tensile test and pure shear test were performed on a universal tensile machine (AGS-X, SHIMADZU, Kyoto, Japan) at room temperature. Each sample was measured in triplicate. The tensile measurement was performed at a crosshead speed of 10 mm min^−1^. The stress with 0 to 10% strain was used to calculate the elastic modulus (E). The stress σ was calculated by the following equation [34].

(1) $$\sigma =\;\frac{F}{WT}$$

where *F* is force, *W* and *T* mean width and thickness of sample. The pure shear test is used to calculate the fracture energy. Two same samples are used at one test, one notched sample and one un-notched sample, and the notched sample is measured at first to get the point at which crack propagation began, while the un-notched sample is measured to get the force-displacement curve. The fracture energy is calculated by the following equation [35].

(2) $$Fracture\;energy\;=\;\frac{\int_{l_{0}}^{l_{c}}Fdl}{WT}$$

where *W* and *T* mean width and thickness of sample, *l_c_* represents critical distance, at which point crack propagation occurs, lo means initial length of sample. 

### 2.5. Rheological Test

The rheological properties of the SA solution with various compositions were analyzed using an AR-G2 stress-controlled rheometer (TA Instrument, New Castle, DE, USA) with a parallel plate geometry (1 mm) at 45 °C. The viscosity of hybrid ink was also measured with different temperatures from 35 °C to 65 °C. The shear rate was varied from 0.01 s^−1^ to 1000 s^−1^. The oscillation frequency measurements were conducted at 35 °C and at a frequency of 1 Hz to measure storage and loss modulus of ink solution. The tan δ values was calculated as [18]:

(3) $$Tan\;\delta =\;\frac{G^{\prime \prime }}{G\prime }$$

where *G′* and *G′′* are storage and loss modulus, respectively. 

### 2.6. Conductivity Test

The hydrogel sample was clipped onto a universal tensile machine (AGS-X, SHIMADZU, Kyoto, Japan) at room temperature around 20 °C. The speed was 10 mm min^−1^. After the hydrogel sample was stretched at certain strain, the resistance was measured by a multi-meter. To limit water evaporation from 3D printed hydrogels, VHB were used to wrap up hydrogel sample during test. To achieve a wearable strain sensor, 3D printed hydrogels covered with VHB were connected to electric wires and placed on a finger with the help of copper tape. To study conductivity of hydrogels at various concentration of ions and SA, calcium alginate (CA)/polyacrylamide(PAAm) hydrogels were fabricated via an injection molding method as previous work [11]. Different concentration of CaCl_2_ (50 mM, 100 mM, 300 mM and 500 mM) were used to soak hydrogels. Three concentrations of alginate (10 mg/mL, 20 mg/mL and 30 mg/mL) and acrylamide (1.69 M, 2.53 M and 3.38 M) were used.

### 2.7. Cytotoxicity Test

The cytotoxicity test of hydrogels was performed as previous method using U87-MG glioblastoma cells (ATCC HTB-14) cultured by Eagle’s Minimum Essential Medium (EMEM) with 10% fetal bovine serum (FBS) and 100 IU ml^−1^ penicillin and streptomycin [13,36]. The hydrogels were soaked in DI water for 8 days, followed by being immersed in the cell cultural medium for 3 days to remove residual monomer and chemicals. After that, the washed hydrogels were put into another cell cultural medium again for 3 days to achieve conditioned cell cultural medium. Following this, 2 × 10^4^ U87-MG cells were seeded onto a 24-well plate and cultured in fresh cell cultural for 3 days. Then the conditioned cell cultural medium was used to culture the cells for another 24 h or 48 h. To evaluate the cytotoxicity of hydrogels, a crystal violet staining method was used. First, cells were fixed by 1 mL of 2% glutaraldehyde in triplicate for 10 min. Then the medium was aspirated and the cells were washed with phosphate-buffered saline (PBS) twice. Then, 1 mL of 0.1% crystal violet was added into the plate and the cells were stained for 40 min. Crystal violet was then washed away by water. Crystal violet was present only in the stained cells. The plate was further drained inversely overnight. The cytotoxicity was evaluated by reading the absorbance at 590 nm of the crystal violet.

In addition, Trypan blue was used to show the cell viability by the previous method [13,36]. Specifically, old media in the 24 well plate were aspirated and 1 mL of Dulbecco’s phosphate-buffered saline (DPBS) was added to wash the adherent cells. Then, DPBS was added into the counting chamber, and the chamber was inserted into a Cellometer Vision Image Cytometry for counting and imaging. 

### 2.8. Swelling Test

The swelling test was performed by soaking four hydrogel samples (400 mg) in DI water for different time periods. The swelling ratio (SR) was defined as [13]:

(4) $$SR=\frac{Ws-Wo}{Wo}\times 100\%\;$$

where *Ws* and *Wo* represent the weight of hydrogels after swelling in DI water in different time periods and the weight of hydrogels before swelling, respectively.

### 2.9. Statistical Analysis 

A one-way analysis of variance (ANOVA) with Fisher’s pair-wise multiple comparison was employed to analyze the data. A *P*-value smaller than 0.05 was considered statistically significant.

## 3. Results and Discussions

### 3.1. Viscosity and Printability

**Figure 2:**
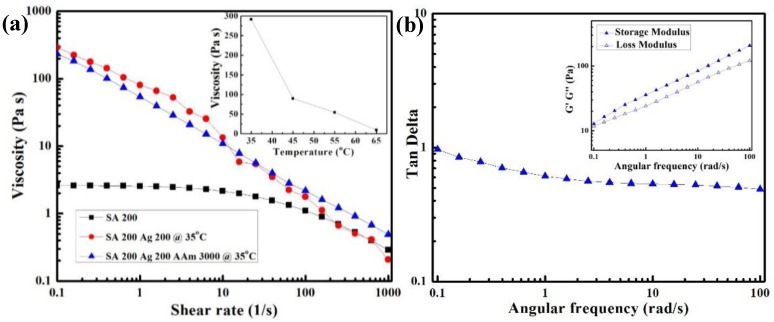
(**a**) Rheological data for ink formulation, the insert: Viscosity of Sodium alginate (SA) 200/Ag 200 with different temperature at shear rate 1/s, and (**b**) Tan δ of the ink solution, the inset: the storage and loss modulus of ink solution.

To achieve 3D printable tough and conductive hydrogels, pre-gel solution was prepared by mixing sodium alginate (SA), agar (Ag), acrylamide (AAm), *N*,*N*’-methylenebis (acrylamide) (MBAA) and Irgacure 2959 into DI water. To well understand printability of hydrogels, the viscosity of pre-gel solution was well characterized by an AR-G2 stress-controlled rheometer. As shown in Figure 2a, the pure SA solution shows a low zero-shear viscosity that is below 30 mPa s that is regarded as minimum solution viscosity for extrusion printing in previous literature, leading to poor shape fidelity [23]. The increased viscosity can help to improve shape fidelity during printing. To improve the shape fidelity of SA, thermo-responsive agar gel solution was added into the 3D printing ink formulation. Compared to SA, agar is a thermo-responsive polymer, which exhibits high viscosity at low temperature. The mixture agar and SA exhibited an extremely higher viscosity at low shear rate and was shear thinning. With temperature decreasing, the viscosity of mixture dramatically increased because of agar gelation (the insert in Figure 2a). After adding a large amount of acrylamide, the printing ink was prepared, and the change of viscous behavior was negligible. Figure 2b showed storage and loss modulus, and tan δ of ink solution (SA 200 mg, Ag 200 mg, and AAm 3000 mg). Tan δ is the ratio of loss modulus to storage modulus. The printing ink solution had a tan δ value below 1, indicating this printing ink is more gel-like than liquid [16].

### 3.2. Mechanical Properties

**Figure 3:**
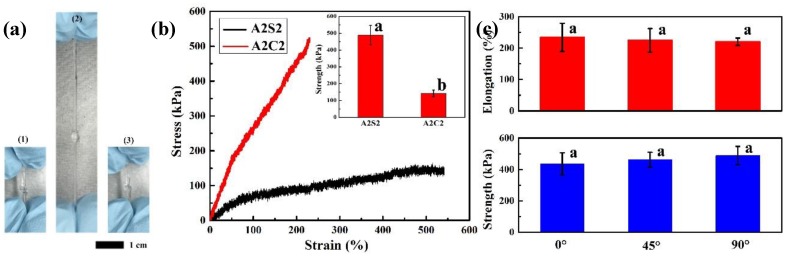
(**a**) Photograph of 3D tough hydrogels, (1) original state, (2) under tension and (3) back to original state, (**b**) stress-strain curve of 3D printed gels with or without CaCl_2_ soaking, the insert is strength of 3D printed gels, means with different letters are statistically different at P < 0.05, (**c**) strength and elongation of 3D printed gels with different printing angles (A2C2), (P > 0.05), means with different letters are statistically different at P < 0.05.

To well investigate mechanical properties of 3D printed hydrogels, tensile test and pure shear test were performed to characterize strength and fracture energy of 3D printed hydrogels. After photopolymerization and calcium chloride soaking, 3D printed tough hydrogels were successfully achieved. As shown in Figure 3a, the 3D printed hydrogels could be easily wrapped into a knot without any damage. After stretching, this knot could return to the original state. This result indicates that the 3D printed tough hydrogels own excellent mechanical properties and good elasticity. The stress-strain curves showed that the 3D printed hydrogels without calcium chloride solution soaking own a weak tensile strength, however, the tensile strength is largely improved after soaking process (as shown in Figure 3b). The improved tensile strength derives from formation of calcium ionically crosslinked alginate network during soaking process. The broken of hydrogels usually contains two sequential steps: initial fracture formation (nucleation) and subsequent fracture propagation (growth) [17]. In A2S2 gels, no nucleation is found due to the agar chain pullout mechanism [37]. With increasing strains, the agar chains pullout from the aggregated agar helical bundles progressively. However, the agar network remains integrated during this process. With addition of alginate chains, the weak entanglements between chains nearly restricts the pullout process of agar. This mechanism allows that the A2S2 gels own relatively long elongation. However, the A2C2 gels showed a distinctive breaking process with a higher strength and smaller elongation. With the addition of the Ca^2+^, the alginate chains are ionically crosslinked. The stress is concentrated on the ionically crosslinked alginate chains and unzip ionically crosslinked alginate chains preferentially leading to breakage of printed hydrogels at relatively low elongation. Therefore, A2C2 gels achieved the higher toughness at 603.22 ± 61.78 kJ m^−3^ while the A2S2 gels depicted a little smaller toughness at 493.27 ± 42.00 kJ m^−3^, although the A2C2 had a larger strength 488.75 ± 58.31 kPa and the A2S2 owned a very small strength at 142.67 ± 19.60 kPa (Table 2). In addition, the mechanical properties of printed gels with different compositions have been also summarized in Table 1 and Figure S2. With increasing amount of alginate and agar, the strength and toughness are improved, however, the elongation is nearly changed due to the existence of calcium crosslinked alginate network. Table S2 and Figure 3c summarized the mechanical properties of the printed gels with various printing infill angles. Three different infill angels including 0°, 45° and 90° were employed in this experiment and the design of 3D printing constructs was presented in Figure S1. The mechanical strength and elongation of these printed gels were similar. Therefore, the presence of calcium crosslinking of alginate network makes the infill have no influence on mechanical properties of printed gels. 

**Figure 4:**
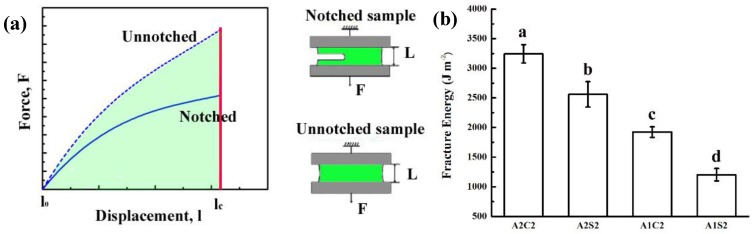
(**a**) Schematic diagram of the pure-shear test for measuring fracture energy of hydrogels, and (**b**) Fracture energy of 3D printed hydrogels, means with different letters are statistically different at P < 0.05.

The pure shear test was used to calculate the fracture energy of 3D printed tough hydrogels. The schematic diagram of the pure-shear test was presented in Figure 4a and detail of measurement was located in experimental section. The fitted stress-strain curves of notched and un-notched hydrogels clearly were presented in Figure S3. According to Equation (2), the fracture energy of 3D printed hydrogels with different compositions were calculated. As shown in Figure 4b, with calcium ionically crosslinked alginate, the fracture energy is largely improved due to increased crosslinking points. When increasing the amount of agar chains, the fracture energy also increases, which demonstrates that higher crosslinking degree leads to higher fracture energy.

**Figure 5:**
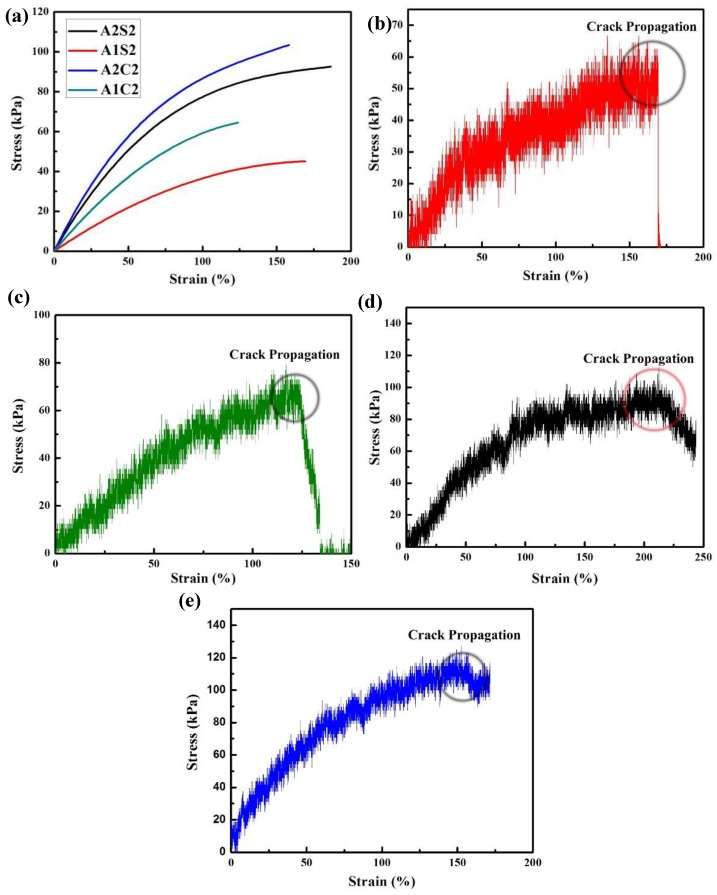
(**a**) Fitted stress-strain curve of notched samples with different compositions, and stress-strain curve of notched sample (**b**) A1S2, (**c**) A1C2, (**d**) A2S2, and (**e**) A2C2.

As shown in Figure 5a, the fitted stress-strain curve of notched samples with various compositions clearly presents that the strength at fracture are both improved with increasing amount of agar and with calcium chloride solution soaking. In addition, we can clearly find that the weak gel (A1C2 and A1S2) crack propagates very quickly and even immediately (Figure 5a,b). That means the notched sample is totally treated in a quick time. However, with increasing agar amount, the crosslinking degree is improved and the notched gel sample of A2S2 is slowly torn (Figure 5c). However, after calcium chloride soaking, the 3D printed tough gels (A2C2) have much slower crack propagation (Figure 5d). That means the combination of agar and calcium alginate (CA) network can greatly prevent the crack propagation. 

### 3.3. Swelling and Cytotoxicity

**Figure 6:**
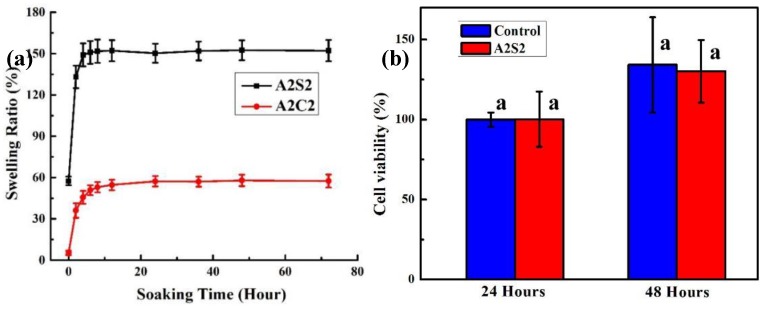
(**a**) Swelling ratio of A2C2 and A2S2 gel, (**b**) Cell viability of U87-MG cells after culturing 24 h and 48 h (P > 0.05), means with different letters are statistically different at P < 0.05.

The swelling properties of printed gels are also systematically investigated. In the soaking process, the water molecules penetrate into polymeric hydrogels resulting in an expansion of polymeric networks and a low concentration region of polymeric chains, which leads to mechanical fracture [11]. Therefore, different chemical structure and crosslinking density can result in different swelling properties. As shown in Figure 6a, the A2S2 gels depict a much larger swelling ability, compared to A2C2 printed gels. The presence of calcium ionically crosslinked alginate not only increases the crosslinking degree, but also restricts the agar and polyacrylamide chains, causing limited swelling ability. 

On the other hand, with increasing amount of agar and calcium crosslinked alginate, the swelling ability is quenched, which demonstrates that the high amount of hard network leads to an inferior swelling ability (Figure S4a and S4b). These results are consistent to the mechanical properties of printed gels. As shown in Figure S4c, the printed gels with different infill angles present exactly the same swelling ability, which demonstrates that the infill angles have no influence on chemical structures and crosslinking degree.

The cytotoxicity of 3D printed hydrogels was also evaluated via U87-MG cells. Figure 6a depicted similar viable cell quantities between the control group and A2S2 hydrogels conditioned group in both 24 and 48 h. These results reveal that the 3D printed hydrogels own high biocompatibility after removing the unreacted acrylamide monomer. Compared to 24 h culture group, the number of viable cells after 48 h culture increased. This result demonstrates that the gels conditioned medium cannot affect cellular reproduction. The Figure S5 presented the live and dead cell images. Cells circled with green were viable, indicating these 3D printed hydrogels own high biocompatibility. After removal of the unreacted acrylamide monomer and other residues, the 3D printed hydrogels own low cytotoxicity with excellent mechanical strength and toughness, which can be considered as a potential candidate in wearable electronics.

### 3.4. Conductivity and Sensors

**Figure 7:**
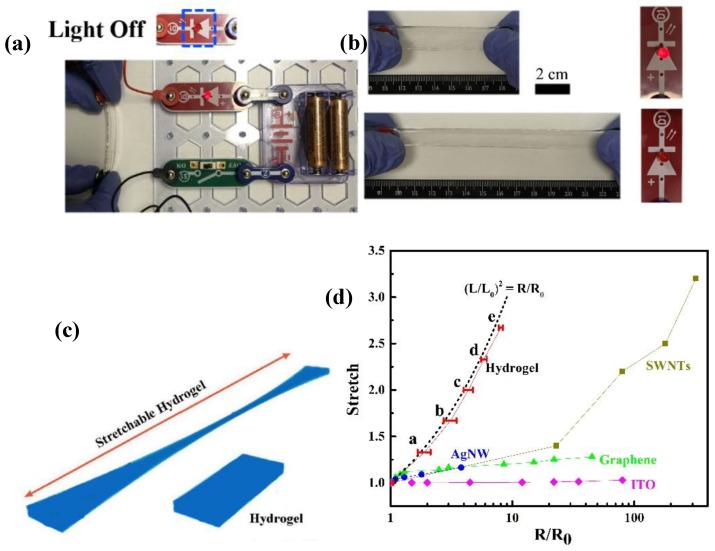
(**a**) Photograph of printed hydrogels connected in the electric circuit, (**b**) photographs of the light changes of elongation connected in the electric circuit, (**c**) schematic diagram of hydrogels when stretching, (**d**) the normalized resistance of printed hydrogels is measured as a function of stretch, means with different letters are statistically different at P < 0.05, plotted against the ideal geometric behavior, and normalized resistance for ITO [40], AgNWs [41], graphene [42], SWNTs [43].

As shown in Figure S6a, high transparency of 3D printed gel was achieved, which allowed these 3D printed hydrogels to detect resistance without affecting optical signals. Due to existence of large amounts of water inside hydrogels, hydrogels offer physical similarity to biotissues, and also own excellent capability to contain lots of ions [38]. By introducing Ca^2+^ and Cl^-^ ions in the 3D printed hydrogels, conductive hydrogels could be used as ionic wires in the circuit. The Figure 7a depicted the bright light when 3D printed conductive hydrogels were connected into the circuit. The light was off, when conductive hydrogels was moved out of circuit. It demonstrates that 3D printed hydrogels are ionically conductive by containing Ca^2+^ and Cl^-^ ions. The resistance of conductive hydrogels is measured via a multi meter. The conductivity of 3D printed hydrogels is around 13.9 mS/cm, which is similar to the conductivity of electrolyte, which demonstrates that the conductivity of conductive hydrogels is close to the conductivity of calcium chloride solution. To well investigate conductivity of hydrogels, calcium alginate (CA)/PAAm double network (DN) hydrogels were fabricated via injection molding method according to our previous work with various concentration of ions, SA and AAm [11]. As shown in Figure S7a, with increasing concentration of CaCl_2_, the conductivity of DN hydrogels enhances. In addition, the conductivity of DN hydrogels with various concentration of CaCl_2_ were close to the that of CaCl_2_ solution with same concentration. These results strongly indicate that conductivity of hydrogels derives from ions. Furthermore, Figure S7b and S7c exhibited no significant change of DN hydrogels’ conductivity with various concentration of SA and AAm. It demonstrates that the polymeric network in DN hydrogels has no effect on conductivity of ionically conductive hydrogels.

Figure 7b showed reduced brightness of the light when the 3D printed hydrogels were stretched, indicating that the stretch largely enhanced the resistance of hydrogels. This result exhibits that the conductivity of these conductive hydrogels is dependent on the strains. When the stretch was released, the brightness of light turned up again. To further investigate the resistance change of 3D printed hydrogels, the resistance change was measured with various stretches. We first assume the 3D printed hydrogels are incompressible and the conductivity is constant during stretching (Figure 7c). The resistance ratio is given by R/R_0_ = (L/L_0_)^2^, where R and R_0_ mean the resistance of the stretched hydrogels and initial hydrogels, respectively. Figure 7d showed that the experimental data of the conductive hydrogels were close to the curve of theoretic equation. The small deviation of experimental data from theoretic curve might be caused by damage in the hydrogels. These results indicate that the measured resistance of the conductive hydrogels is reasonable. By comparison with other electronic conductors like indium tin oxide (ITO), silver nanowires (AgNWs), graphene, single-wall nanotubes (SWNTs), the conductive hydrogels, ionic conductors, owned lower conductivity than these mentioned electronic conductors. However, when high transmittance and stretchability are necessary, these conductive, transparent and stretchable hydrogels have specific advantages. At high stretch, these hydrogels had lower sheet resistance than these above mentioned electronic conductors. This result is consistent with previous literature [39]. 

**Figure 8:**
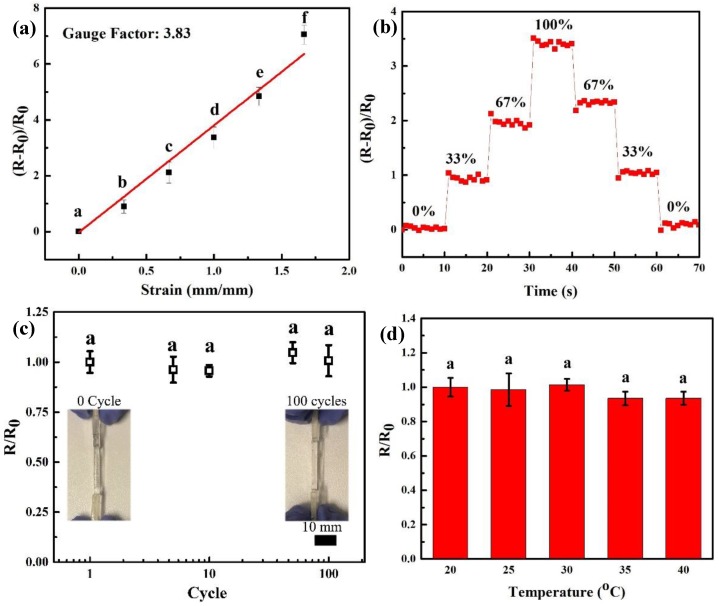
(**a**) The dependence of sensing sensitivity of conductive hydrogels with the applied strain. The strain sensitivity (S) can be defined as the slope of resistance change rate (*ΔR*/*R_0_*) versus applied strain (λ), formulized as *S* = δ(*ΔR*/*R_0_*)/δλ, means with different letters are statistically different at P < 0.05, (**b**) The relative resistance changes vs time when a loading−unloading cycle of conductive hydrogels at different strains, (**c**) the resistance ratio of conductive hydrogels as a function of fatigue cycle number (P > 0.05), means with different letters are statistically different at P < 0.05, and (**d**) the resistance ratio of conductive hydrogels as a function of temperature (P > 0.05), means with different letters are statistically different at P < 0.05.

As depicted in Figure 8a, the resistant change of these conductive hydrogels showed a good linear correlation to strain in a range of 0–1.5, which indicates that the conductive hydrogels own a relatively large sensing range. The strain sensitivity of conductive hydrogels can be defined as the slope of resistance change rate (*R*−*R_0_*/*R_0_*=*ΔR*/*R_0_*) versus applied strain (λ), formulized as *S*=δ(*ΔR*/*R_0_*)/δλ [38]. A gauge factor (3.83) was achieved via these conductive hydrogels that is superior to previously reported hydrogel-based strain sensor (0.478) [38]. This result demonstrates the conductive hydrogels exhibit a high sensing sensitivity. As shown in Figure 8b, the relative resistance change of these conductive hydrogels was exhibited during a step-by-step loading-unloading cycle at different strains. This curve clearly showed the relative resistance of conductive hydrogels had a step-like trend. In addition, the relative resistance directly increased or decreased when conductive hydrogels were stretched or released to certain strain. It is meaningful that conductive hydrogels have quick response ability, because no hysteresis was found during strain change. Moreover, a good sensing stability of conductive hydrogels was also observed, since the relative resistance was kept stable during load-holding or unload-holding period at different strain. As a wearable strain sensor, it is also important for the conductive hydrogels to own high stability. The Figure 8c showed the relative resistance change for 100 tensile cycles under 10% strain. The resistance of the conductive hydrogels was similar to the original level and these hydrogels showed no visible damage or delamination after 100 cycles of 10% strain. These results demonstrate that the conductive hydrogels have superior stability in sensing. The Figure 8d showed no obvious relative resistance change in temperature range of 20 to 40 °C, which means that these conductive hydrogels own high reliability when they are attached onto the human body.

**Figure 9:**
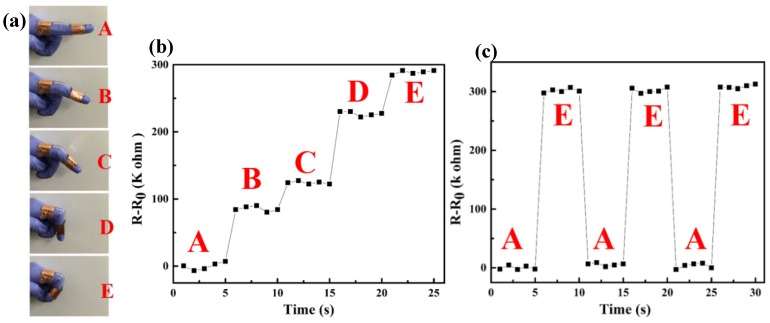
(**a**) Photographs of finger bending, (**b**) resistance change when finger bending and (**c**) repeated response of the resistive strain sensor.

In addition, we developed a wearable resistive strain sensor by these 3D printed conductive hydrogels and fixed it onto an index finger by copper tapes to monitor finger bending (Figure 9a). When the index finger bended step-by-step, the relative resistance change of this sensor rose up in a step-like trend (Figure 9b), which is similar to results in Figure 8b. The relative resistance of this wearable resistive strain sensor directly enhanced without hysteresis after the finger bending to a gesture, which exhibits fast response ability. During gesture-holding process of the index finger, the resistance of the sensor could remain at a constant, which shows the good sensing stability of this wearable resistive strain sensor. The Figure 9c also depicted a repeatable response during finger bending, which demonstrated that this wearable strain sensor has a good sensing stability in repeated usage. These results indicate that this conductive hydrogels-based strain sensor can be applied for human motion monitoring with high sensing sensitivity and stability.

## 4. Conclusion

In summary, a tough and conductive hydrogel was developed by 3D printing technology. The combination of alginate and agar guarantee high ink viscosity resulting in high printing precision. A double network structure that combined covalent crosslinking and Ca^2+^-alginate coordination was employed to achieve conductive, transparent and stretchable hydrogels. The results demonstrated that the double network structure affords a smooth stress-transfer and recoverable energy dissipation to gift the hydrogels with superior mechanical strength and toughness. In addition, the 3D printed hydrogels containing a large amount of water that dissolves calcium ions, could work as ionic conductors. Conductive hydrogels depict quick, steady and repeated deformation toward strain to change the ionic transport, leading to rapid sensing response, high sensing stability and strain sensitivity. A wearable resistive strain sensor was fabricated connecting one 3D printed hydrogel film with conductive tape, which can rapidly and precisely detect the joint motions of finger bending. These results support the possibility for developing conductive, transparent, and stretchable hydrogels as wearable resistive strain sensor for human motion detection or sensory skin employed in a soft robot.

## Figures and Tables

**Table 1:** The formula of printing ink in 10 mL Deionized (DI) water.

Sample	Agar (mg)	Sodium Alginate (mg)	Irgacure 2959 (mg)	Acrylamide (mg)	MBAA (mg)	Concentration of CaCl_2_ (mM)
A1C2	100	200	90	3000	3	100
A2C2	200	200	90	3000	3	100
A3C2	300	200	90	3000	3	100
A2C1	200	100	90	3000	3	100
A2C3	200	300	90	3000	3	100
A1S2	100	200	90	3000	3	N/A
A2S2	200	200	90	3000	3	N/A

**Table 2:** The mechanical properties of the printed hydrogels with different composition.

Sample	Young’s Modulus (kPa)	Strength (kPa)	Elongation (%)	Toughness (kJ m^−3^)
A1C2	26.74 ± 4.95	385.56 ± 31.10	223.63 ± 62.96	479.71 ± 150.97
A2C2	38.14 ± 2.99	488.75 ± 58.31	220.30 ± 11.74	603.22 ± 61.78
A3C2	55.45 ± 9.33	744.57 ± 144.17	235.78 ± 31.95	1049.77 ± 241.21
A2C1	17.14 ± 4.63	283.33 ± 31.07	215.44 ± 12.10	362.01 ± 31.26
A2C3	41.20 ± 3.61	596.84 ± 28.43	218.54 ± 24.81	762.02 ± 43.76
A2S2	16.29 ± 0.54	142.67 ± 19.60	566.38 ± 23.47	493.27 ± 42.00

## Supplementary Materials

The following are available online at https://www.mdpi.com/2073-4360/11/11/1873/s1, Figure S1: Design of 3D printed hydrogels with different infill angles (a) 0°, (b) 45° and (c) 90°, Figure S2: (a) Stress-Stain curve of 3D printed gel with different alginate concentration, (b) strength of 3D printed gels with different agar content (P > 0.05), means with different letters are statistically different at P < 0.05, (c) Stress-stain curve of 3D printed gel with different agar concentration, and (d) strength of 3D printed gels with alginate content (P > 0.05), means with different letters are statistically different at P < 0.05, Figure S3: The fitted stress-strain curve of both notched and unnotched sample (a) A1S2, (b) A1C2, (c) A2S2, and (d) A2C2, Figure S4: (a) swelling ratio of gel with different agar content, (b) swelling ratio of gel with different alginate content, and (c) Swelling ratio of A2C2 gel with different infill method, Figure S5: Live and dead cell image (a) control, and (b) A2S2, Figure S6: High transparency and conductivity of 3D printed hydrogels, Figure S7: (a) Conductivity of hydrogels (sodium alginate (SA) 200 mg and acrylamide (AAm) 1200 mg) by injection molding method with various concentration of calcium chloride, means with different letters are statistically different at P < 0.05, (b) Conductivity of hydrogels (AAm 1200 mg and CaCl2 100 mM) by injection molding method various alginate content, (P > 0.05), means with different letters are statistically different at P < 0.05, and (c) Conductivity of hydrogels (SA 200 mg and CaCl2 100 mM) by injection molding method various concentration of acrylamide, (P > 0.05), means with different letters are statistically different at P < 0.05, Table S1: the formula of printing ink in 10 mL DI water, Table S2: the mechanical properties of printed hydrogels with different printing parameter.
