# Supplementary material for: Ultrasensitive Wearable Strain Sensors of 3D Printing Tough and Conductive Hydrogels
Source: Polymers (Basel). 2019 Nov 13;11(11):1873. doi: 10.3390/polym11111873 (PMC6918434; doi:10.3390/polym11111873)
Supplement: Supplementary file 1 [file polymers-11-01873-s001.pdf]

Support Information

Ultrasensitive Wearable Strain Sensors of 3D Printing Tough and Conductive Hydrogels

Jilong Wang [1], Yan Liu [1], Siheng Su [2], Junhua Wei [3], Syed Ehsanur Rahman [3], Fuda Ning [4], Gordon Christopher [3], Weilong Cong [5] and Jingjing Qiu [3,*]

1 Key Laboratory of Textile Science & Technology of Ministry of Education, College of Textiles, Donghua University, Shanghai 201620, PR China; jilong.wang@dhu.edu.cn (J.W.); yan.liu@mail.dhu.edu.cn (Y.L.)
2 Department of Mechanical Engineering, California State University Fullerton, Fullerton, CA 92831, United States; ssu@fullerton.edu
3 Department of Mechanical Engineering, Texas Tech University, 2500 Broadway, Lubbock. TX 79409, United States; junhua5wei@gmail.com (J.W.); syed.rahman@anton-paar.com (S.E.R.); gordon.christopher@ttu.edu (G.C)
4 Department of Systems Science and Industrial Engineering, State University of New York at Binghamton, Binghamton, NY 13902, United State; fning@binghamton.edu
5 Department of Industrial Engineering, Texas Tech University, 2500 Broadway, Lubbock. TX 79409, United States; weilong.cong@ttu.edu
* Correspondence: jenny.qiu@ttu.edu.

Table S1, the formula of printing ink in 10 ml DI water

Sample	Agar (mg)	Alginate (mg)	Irgacure 2959 (mg)	Acrylamide (mg)	MBAA (mg)	Concentration of $CaCl_2$ (mM)
A1C2	100	200	90	3000	3	100
A2C2	200	200	90	3000	3	100
A3C2	300	200	90	3000	3	100
A2C1	200	100	90	3000	3	100
A2C3	200	300	90	3000	3	100
A1S2	100	200	90	3000	3	N/A
A2S2	200	200	90	3000	3	N/A

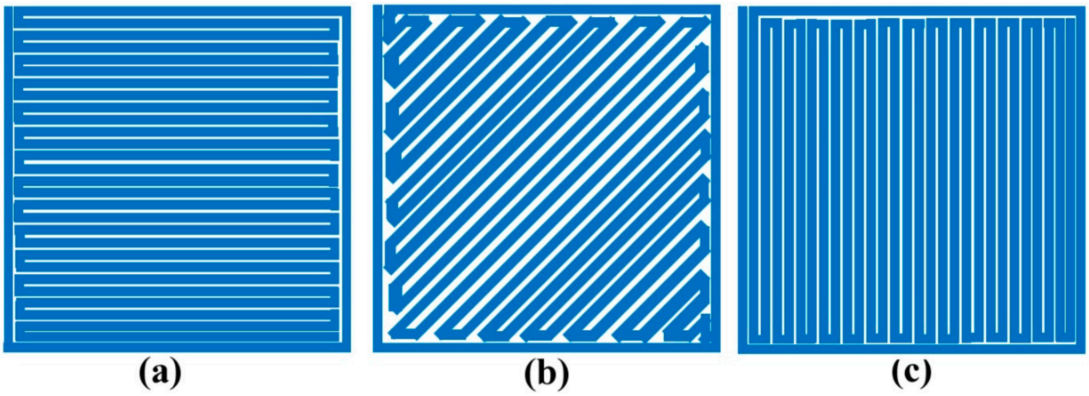

Figure S1 Design of 3D printed hydrogels with different infill angles (a) 0°, (b) 45° and (c) 90°.

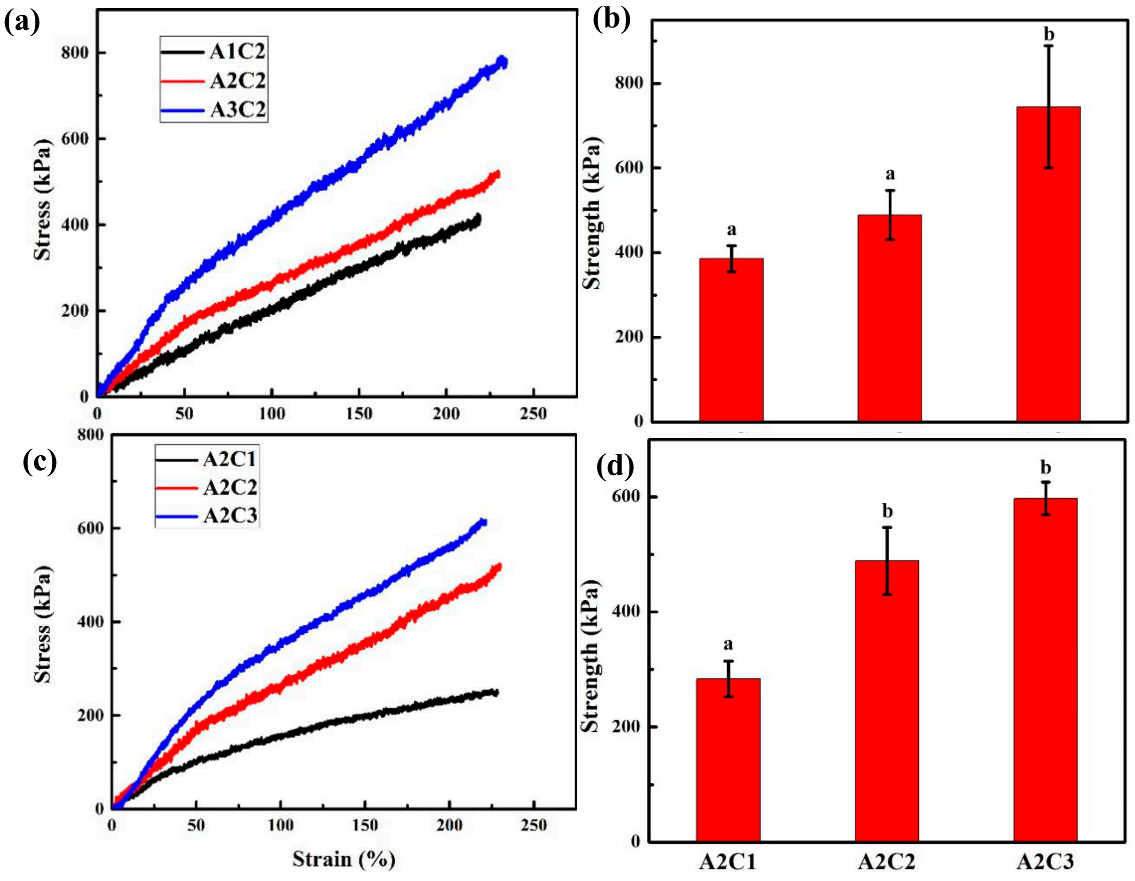

Figure S2, (**a**) Stress-Stain curve of 3D printed gel with different alginate concentration, (**b**) strength of 3D printed gels with different agar content (P＞0.05), means with different letters are statistically different at P＜0.05, (**c**) Stress-stain curve of 3D printed gel with different agar concentration, and (**d**) strength of 3D printed gels with alginate content (P＞0.05), means with different letters are statistically different at P＜0.05

Table S2 the mechanical properties of printed hydrogels with different printing parameter

Sample	Printing Temperature (°C)	Printing Infill (°)	Young's modulus (kPa)	Strength (kPa)	Elongation (%)	Toughness (kJ m^{-3})
A2C2	55	0	30.89 ± 9.46	434.74 ± 71.10	234.26 ± 44.94	611.45 ± 197.20
A2C2	55	45	34.21 ± 3.26	461.78 ± 47.50	224.79 ± 37.75	637.89 ± 134.19
A2C2	55	90	38.14 ± 2.99	488.75 ± 58.31	220.30 ± 11.74	603.22 ± 61.78

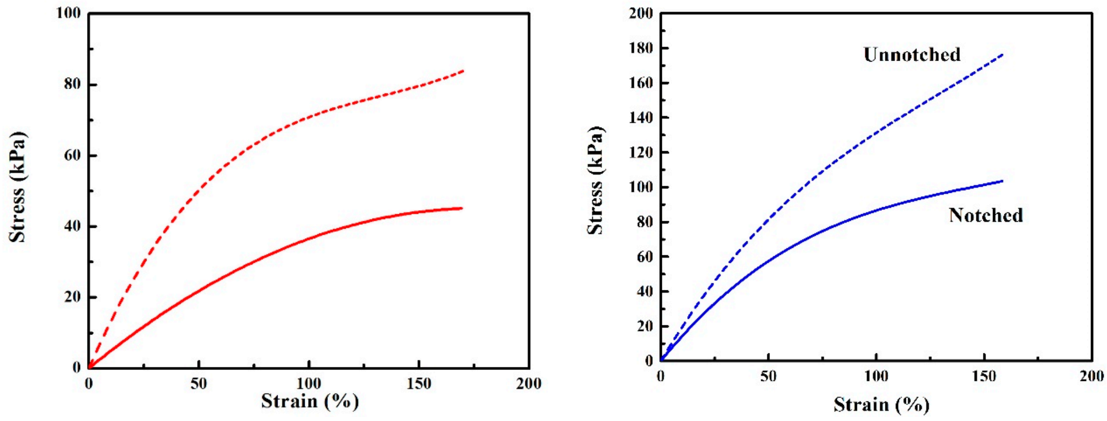

(a) (b)

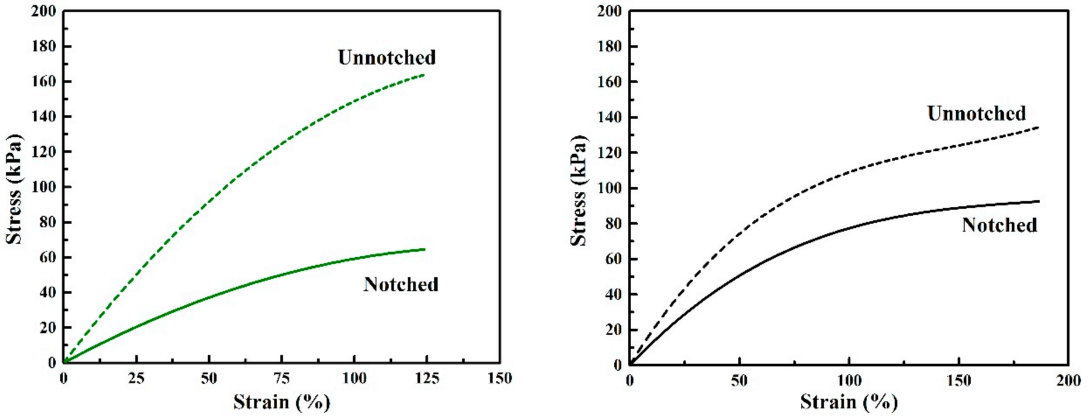

(c) (d)

Figure S3 The fitted stress-strain curve of both notched and unnotched sample (**a**) A1S2, (**b**) A1C2, (**c**) A2S2, and (**d**) A2C2.

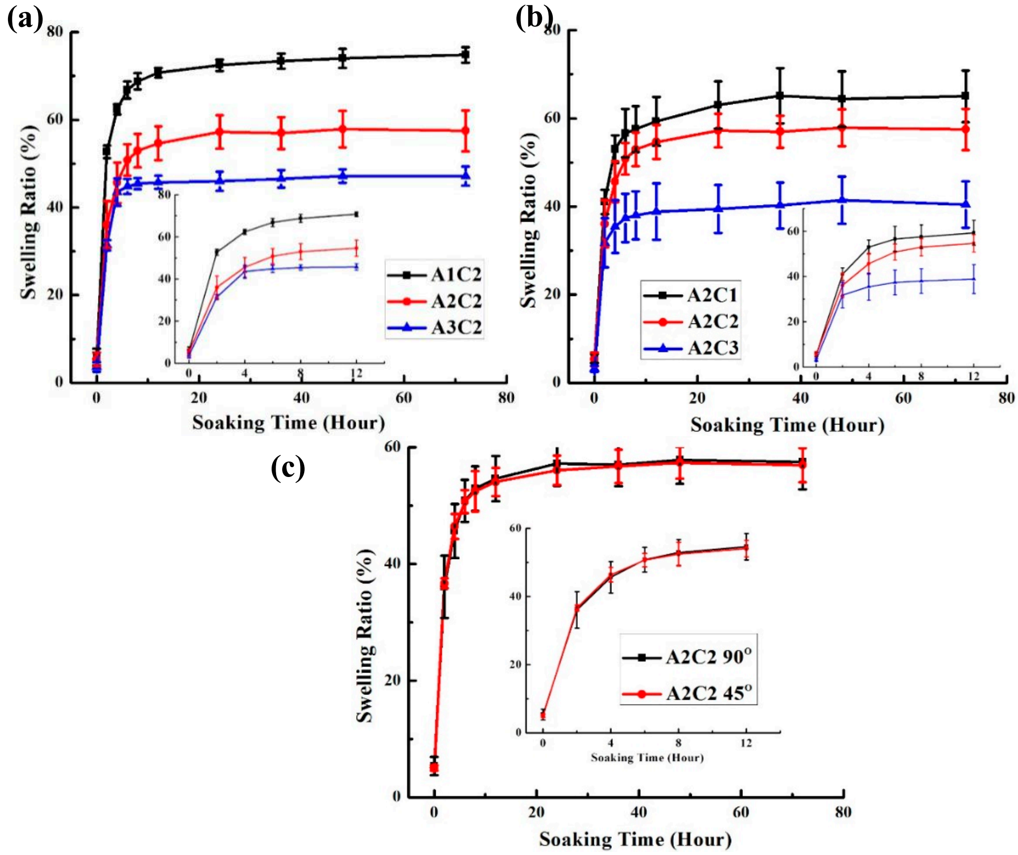

Figure S4 (**a**) swelling ratio of gel with different agar content, (**b**) swelling ratio of gel with different alginate content, and (**c**) Swelling ratio of A2C2 gel with different infill method.

(a) 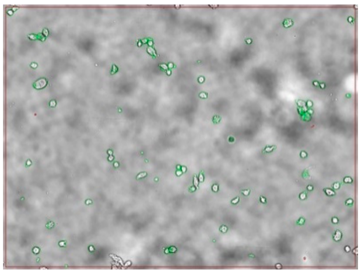 (b) 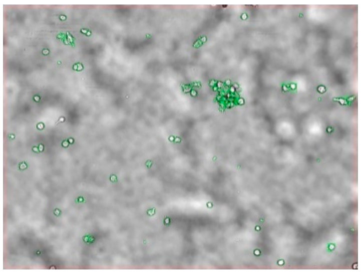

Figure S5 Live and dead cell image (**a**) control, and (**b**) A2S2

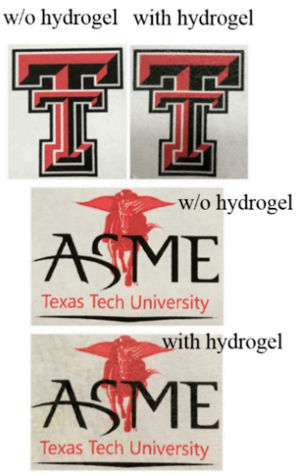

Figure S6 High transparency and conductivity of 3D printed hydrogels.

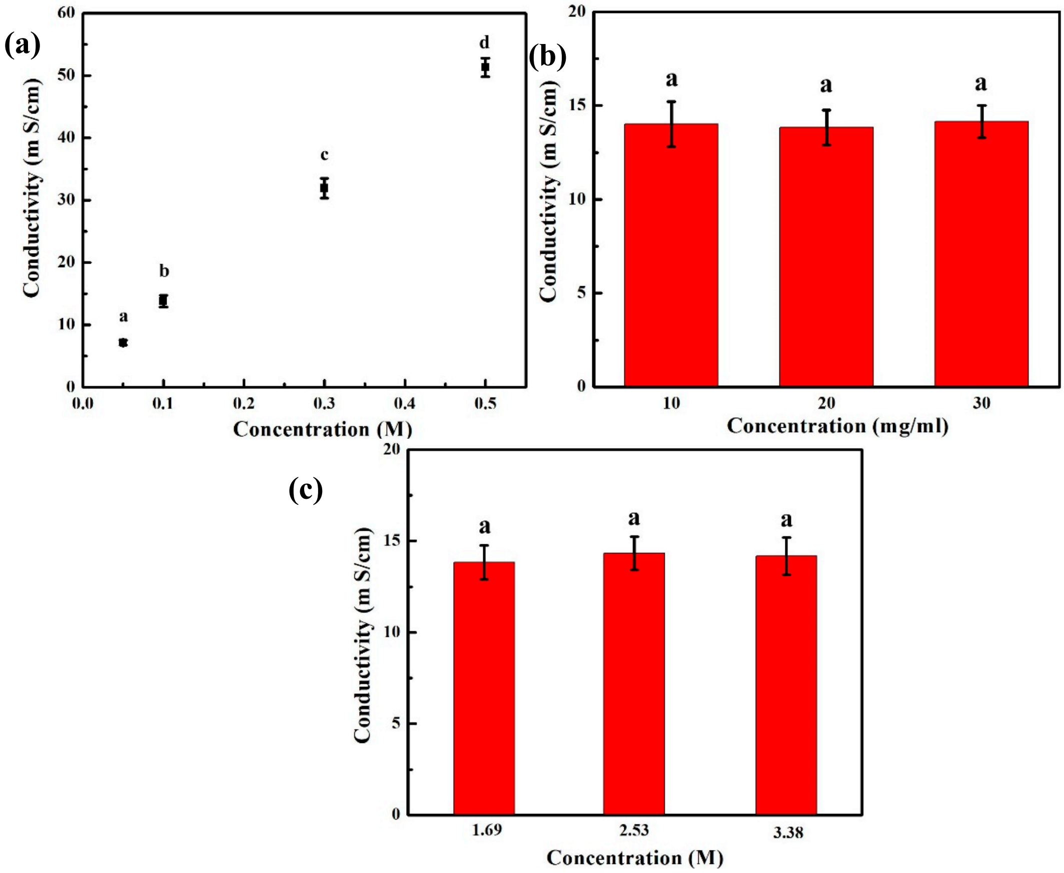

Figure S7 (**a**) Conductivity of hydrogels (sodium alginate (SA) 200 mg and acrylamide (AAm) 1200 mg) by injection molding method with various concentration of calcium chloride, means with different letters are statistically different at $P<0.05$, (**b**) Conductivity of hydrogels (AAm 1200 mg and $CaCl_2$ 100 mM) by injection molding method various alginate content, ($P>0.05$), means with different letters are statistically different at $P<0.05$, and (**c**) Conductivity of hydrogels (SA 200 mg and $CaCl_2$ 100 mM) by injection molding method various concentration of acrylamide, ($P>0.05$), means with different letters are statistically different at $P<0.05$.
